# Application of Proteomics in Cancer: Recent Trends and Approaches for Biomarkers Discovery

**DOI:** 10.3389/fmed.2021.747333

**Published:** 2021-09-22

**Authors:** Yang Woo Kwon, Han-Seul Jo, Sungwon Bae, Youngsuk Seo, Parkyong Song, Minseok Song, Jong Hyuk Yoon

**Affiliations:** ^1^Neurodegenerative Diseases Research Group, Korea Brain Research Institute, Daegu, South Korea; ^2^Department of Convergence Medicine, Pusan National University School of Medicine, Yangsan, South Korea; ^3^Department of Life Sciences, Yeungnam University, Gyeongsan, South Korea

**Keywords:** cancer, proteomics, multi-omics, biomarkers, translational research

## Abstract

Proteomics has become an important field in molecular sciences, as it provides valuable information on the identity, expression levels, and modification of proteins. For example, cancer proteomics unraveled key information in mechanistic studies on tumor growth and metastasis, which has contributed to the identification of clinically applicable biomarkers as well as therapeutic targets. Several cancer proteome databases have been established and are being shared worldwide. Importantly, the integration of proteomics studies with other omics is providing extensive data related to molecular mechanisms and target modulators. These data may be analyzed and processed through bioinformatic pipelines to obtain useful information. The purpose of this review is to provide an overview of cancer proteomics and recent advances in proteomic techniques. In particular, we aim to offer insights into current proteomics studies of brain cancer, in which proteomic applications are in a relatively early stage. This review covers applications of proteomics from the discovery of biomarkers to the characterization of molecular mechanisms through advances in technology. Moreover, it addresses global trends in proteomics approaches for translational research. As a core method in translational research, the continued development of this field is expected to provide valuable information at a scale beyond that previously seen.

## Introduction

Proteomics is the study of the entire set of proteins expressed in a cell, tissue, or individual (1, 2). With the advent of Mass spectrometry (MS)-based protein analysis technology, large-scale protein analysis has now become widely used (3–5). Proteomics involves a wide range of processes such as protein expression profiling, protein modifications, protein-protein interactions, protein structure, and protein function (6, 7). The results obtained from such tasks can be used to decipher disease processes, provide diagnosis and prognosis of diseases, aid in drug development, and deliver the basis for biological discovery (8–11). With the development of proteomics technology and its application to various diseases, especially cancer, significant progress has been made in identifying clinically applicable biomarkers and new therapeutic targets (12–14).

The proteomics approach has become popular in cancer studies. Proteomics-based technologies have enabled the identification of potential biomarkers and protein expression patterns that can be used to assess tumor prognosis, prediction, tumor classification, and to identify potential responders for specific therapies. This information can be obtained from different types of samples and be used to develop cancer therapeutics (15–18). In addition, in order to understand the basic biology of cancer, proteomics techniques have been utilized to understand how the signaling pathways in tumor cells are altered, improving our understanding of how to target various pathways for cancer therapy (19–22). As a result, cancer proteome databases have been created, and massive data sets have been collected and integrated with cancer molecular biology data, as well as clinical information.

Over recent years, multi-Omics approaches using patient samples have been used worldwide in translational research. A multi-Omics analysis is a comprehensive and integrated analysis of combined data generated from various omics approaches, including proteomics, genomics, transcriptomics, and metabolomics (23). Multi-Omics analyses can produce large-scale datasets compared to a single analysis, and provides valuable information on the pathophysiology of diseases caused by complex events, thereby making a significant contribution to the diagnosis of diseases and the development of treatments (24–27). Therefore, the continual use of Omics approaches that aggregates multiple Omics data sets will likely have a significant impact on translational research, including in cancer biology, and will likely be the basis for the study of various diseases going forward.

In this paper, we will provide an overview of different cancer proteomics approaches. We will also discuss the use of proteomics technology in a variety of cancers and global trends in the proteomics approaches mentioned above in translational research across the characterization of molecular mechanisms.

## General Analytical Strategies of Proteomics

**Figure 1:**
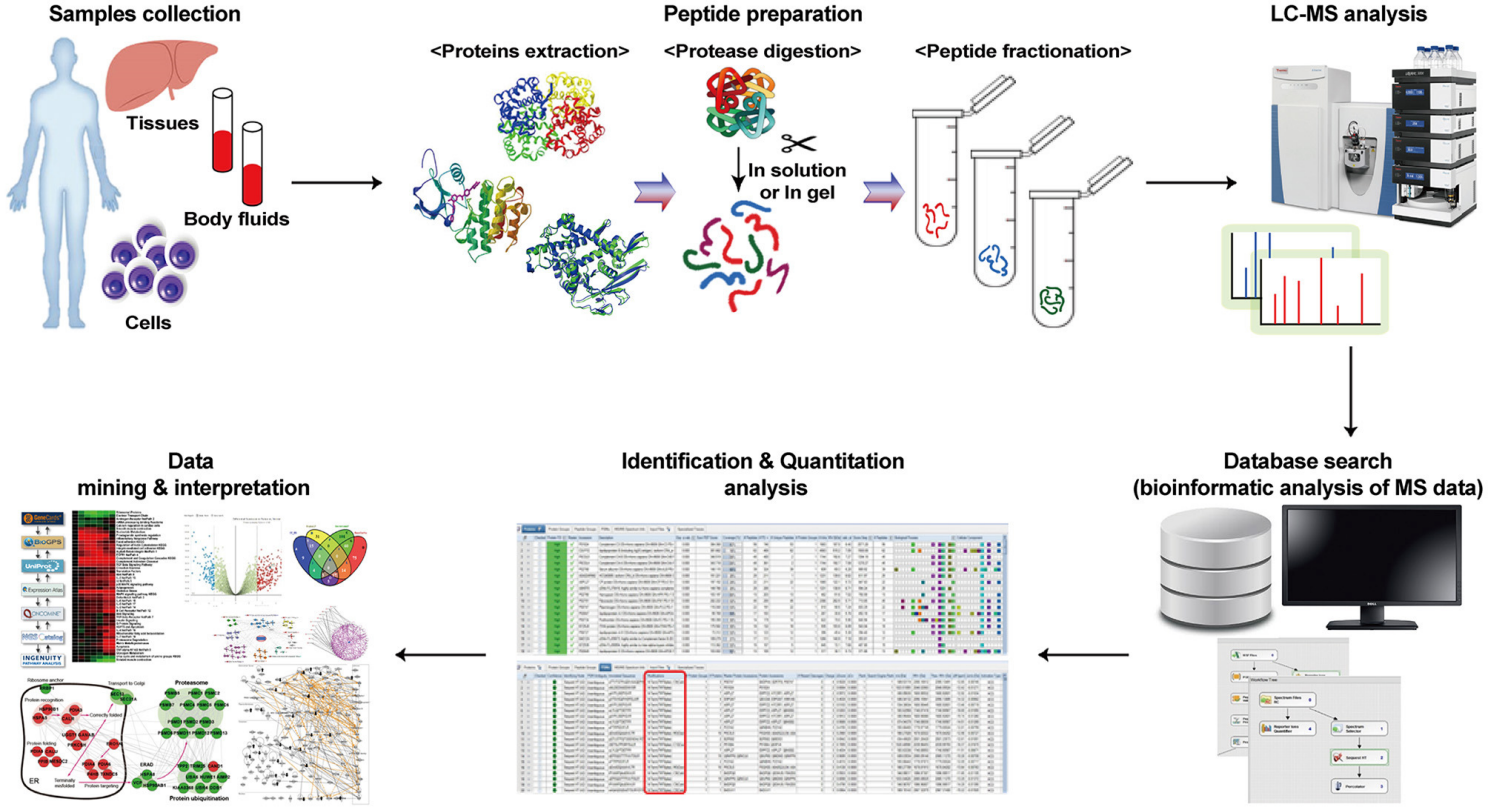
Workflow of the proteomics investigation. Proteomics exhibit many proteins by peptide preparation, analysis using mass spectrometry, and interpretation of peptide data through existing databases.

Analytical platforms for proteomics have been developed to identify the entire set of proteins in organisms and to uncover qualitative and quantitative protein variations upon diverse environmental changes. In addition, comprehensive research on proteins has become possible by building an amino acid sequence database on the composition of proteins (1). Generally, a proteomic analysis consists of the following steps: (1) protein extraction, (2) protease digestion, (3) peptide fractionation, and (4) LC-MS analysis (Figure 1). Initially, proteins are extracted and purified from tissue or cell lysates by centrifugation and filtration. Then, the protein mixture is typically separated by two-dimensional gel electrophoresis to reduce sample complexity. Total proteins can be identified by LC-MS analysis of their peptides, which are produced by enzymatic (usually tryptic) digestion, and the data are interpreted using a proteome database (1, 28–31).

An attractive part of the proteomics field is its ability to reveal novel biomarkers of diseases. For example, as cancer progresses, changes in protein profiles and differences in protein distribution both in tissues and body fluids such as blood can be examined through quantitative analysis. Proteomics enable the simultaneous qualitative and quantitative profiling of numerous proteins. Liquid chromatography-mass spectrometry (LC/MS) is a key technique that obtains high-resolution spectra of mixed peptides, allowing the discovery of sensitive and specific biomarkers associated with cancer (31). The high-throughput technologies based on this technique enable semi-quantitative and quantitative analyses (32). For the quantitative study of proteins, label-based and label-free approaches are widely used in clinical research (Table 1). Label-based quantitation strategies allow the quantitative and qualitative analysis of proteins in a sample. The methods consist in using stable isotope labeling of compound markers such as amino acids to tag proteins or peptides. The samples containing the tagged proteins are then compared with control proteins tagged with isotope-free markers. These methods have the advantage of minimizing disparities between individually handled samples (33–35). However, proteins may be partially labeled and the reagents are expensive. In proteomics, the common labeling methods are SILAC, ICAT, TMT, and iTRAC. SILAC has the least experimental variability because it is a metabolic labeling method; the isotope reagent is used in the initial step of sample preparation (i.e., cell culture) and the labeled proteins are generated through metabolic processes (33). ICAT is a chemical labeling technique that uses a reagent consisting of a functional group that targets cysteinyl residues, a deuterium atom-based linker region, and a biotin group for protein purification. Sample complexity is significantly reduced through affinity-based extraction of labeled proteins (36). Isobaric labeling methods using TMT and iTRAC need tandem MS techniques. Labeling reagents containing reporter ions are produced under tandem MS, and their amount is proportional to that of tagged peptides, resulting in the quantitation of proteins (34, 35). In contrast to label-based strategies, label-free quantitation approaches using MRM or SWATH are straightforward without labeling steps, which is suitable for large-scale studies. Label-free proteins are quantified based on the signal intensities or spectral counts of peptides unique to them, which are obtained from the MS analysis. The recent development of high-resolution mass spectrometers has led to advances in label-free quantitation for proteomics. Label-free quantitation is easy to use, yields highly reproducible results in biochemical experiments, and is reliable when many statistical verifications are required (37–40). The amount of protein can also be analyzed using antibody arrays such as the ELISA. This is a semi-quantitative and quantitative analysis in which capture antibodies are immobilized on a solid surface such as a nitrocellulose membrane, glass slide, silicone, or beads. Then, the interaction between the antibody and its target protein is detected. However, it is not a discovery-oriented method, and it is limited to the detection of usable and compatible proteins (41).

**Table 1:** Types of methods for quantitative proteomics.

**Proteomics method**	**Quantification method**	**Type**	**Principle**	**Advantages**	**Disadvantages**
LC/MS-based proteomics	Labeling	- ICAT - iTRAQ - SILAC - TMT	Isotopic labeling used for quantitative proteomics by MS using chemical labeling reagents	- Depth of field across proteomics - Identify high and low copy gene products - Large-scale analysis of complex components - High accuracy of quantification	- Different ionization efficiency of different samples - Comparisons of varying sensitivity and resolution - Non-native state due to chemical labeling
	Label-free	- MRM - SWATH	Method for relatively quantifying differences in concentration between independent samples using MS	- Reflect the native state without the chemical treatment process - Reduce the margin of error - Reliable for multiple statistical verifications - Relatively low cost	- The analysis system is complex - Calculations take a lot of time - Relatively low accuracy of quantification compared to the labeling method - Multiplexed analysis not possible

*LC/MS: liquid chromatography mass spectrometry; ICAT, isotope-coded affinity tag; iTRAQ, isobaric tags for relative and absolute quantitation; SILAC, stable isotope labeling by amino acids in cell culture; TMT, tandem mass tag; MRM, multiple reaction monitoring; SWATH, sequential window acquisition of all theoretical fragment ion spectra*.

Qualitative and quantitative protein analyses are very important for understanding biological phenomena and discovering molecular biomarkers of diseases. To diagnose cancer and other diseases using proteomics, minute quantitative and state alterations in the expression of specific proteins need to be detected. Since protein profiles may differ between patients, accurate tools for sample selection, analysis methods and data interpretation need be established to identify relevant protein alterations. Early cancer diagnosis and the discovery of novel potential biomarkers require advances in statistical analyses along with technologies capable of detecting and tracking small protein alterations with high accuracy, reproducibility, and analytical throughput.

## Application of Proteomics in Cancers

Cancer involves aberrant cell proliferation, in which the cell cycle of the normal cell becomes dysregulated due to a variety of genetic alterations. Cancer can occur in any tissue of the body and is characterized by its ability to invade or spread to other tissues and organs (42–44). In particular, malignant tumors not only grow rapidly and metastasize to various other tissues but can also develop resistance to the drugs used in treatment, thereby threatening the life of patients (45–47). Proteomics has emerged as an important research tool for exploring the biological changes in cancer. Based on proteomics technology, key information such as protein targets and signaling pathways related to the growth and metastasis of cancer cells have been identified.

## Cancer Growth

As fundamental role of cancer therapeutics is inhibition of aberrant cell growth, proteomics-based approaches can play a decisive role in discovering specific biomarkers for growth of cancer. Recently, isobaric labeling TMT proteomics has been used to study hepatitis B virus related hepatocellular carcinoma (HCC) using patient samples with liver tumors and adjacent healthy tissue. Phosphoproteomic approaches elucidated that PYCR2 and ADH1A are related to metabolic reprogramming in HCC, phosphorylation of ALDOA promotes glycolysis and proliferation in CTNNB1-mutated HCC cells (48). This study has provided mechanistic insight on how to develop effective therapies for the clinical treatment of HCC. Genetically engineered mouse models and primary pancreatic epithelial cells have been developed to perform transcriptomics, proteomics, and metabolic analysis in pancreatic cancer. The study shows that LKB1 in primary pancreatic epithelial cells regulates pathways associated with glycolysis, serine metabolism, and DNA methylation using TMT labeling proteomics and proteomic dataset, suggesting that these may regulate the growth of pancreatic cancer cells. Furthermore, it found mechanism that loss of LKB1 and the activation of KRAS seen in oncogenicity is facilitated with the mTOR-dependent pathway (49). These findings comprehend biological mechanism as regulating the growth of cancer and highlight novel proteomics approaches to analyzing genes, proteins, and metabolites for discovering new cancer therapeutics.

An understanding of the tumor and stromal compartments is vital for understanding the growth that occurs in cancer. In tumor tissues, cancer cells interact with the microenvironment, including cancer-associated fibroblast (CAF). Through the proteomics approach, a major metabolic modulator of CAF has been identified. Using a label-free proteomic workflow, the differences in protein expression between the tumor and the stromal compartment were elucidated and confirmed that NNMT expression was increased in omental metastases of patients. The expression of NNMT in the stroma regulates histone methylation and subsequent transcriptional changes. These are critical for the CAF phenotype, enhancing migration and proliferation in ovarian cancer. In support of these findings, it was also shown that inhibiting NNMT expression in CAFs suppressed tumor growth in *in vivo* experiments, demonstrating the advantages that proteomics can be used to determine the disease phenotypes (50). These studies, by identifying proteins and pathways related to cancer growth and its environment, provide not only the potential to develop effective therapies but also bioinformatic resources to aid basic research.

## Metastasis

The diversity of different cancers and the metastasis that occurs during cancer progression are severe obstacles for the successful development of therapeutics (51–53). In particular, metastasis is the most common characteristic of malignant cancers; however, the precise mechanism by which the metastatic cascade occurs is not clearly defined. Recently, several proteomics studies have been performed in an attempt to uncover the cause of the increased metastasis seen in cancer. In one such study, using several Omics like transcriptomics, proteomics, and phospho-proteomics to examine a patient-derived xenograft mouse model, TMT labeling analysis revealed that an increase in stress hormone levels during breast cancer progression was found to cause an increase in the activity of the glucocorticoid receptor (GR) at metastatic regions ultimately reducing the survival rate. Furthermore, it was found that the increased GR activity was implicated in the activation of multiple processes in metastasis and in the elevated expression levels of the kinase ROR1, both of which correlated with reduced survival. In support of these findings, the depletion of ROR1 reduced metastatic growth and extended the survival rate in preclinical models (54).

Lignitto et al. detected increased expression of Bach1, a pro-metastatic transcription factor, *via* a multi-Omics analysis of the transcriptome and proteome. In lung adenocarcinoma, the loss of keap1 and subsequent Nrf2 activation-induced metastasis through the accumulation of Bach1, and this process was related to a reduction in the survival rate of patients with lung cancer in a heme oxygenase-1-dependent manner. Nrf2 was shown to suppress the Fbxo22-mediated degradation of Bach1 in a heme oxygenase-1 dependent manner, suggesting that inhibition of heme oxygenase-1 could be an effective therapeutic strategy for preventing lung cancer metastasis (55). Such an integrated approach with TMT proteomics analysis defines the role of target molecules in cancer metastasis, providing valid information for diagnosis, prognosis, and therapies.

## Drug Resistance

Cancer can recur despite treatments such as surgery and chemotherapy, suggesting that recurrent cancer contains cells that are resistant to anti-cancer drugs (56, 57). The proteomics approach can be employed to identify the characteristics of drug-resistant cancer cells and discover targets that can overcome drug resistance that develops during anti-cancer treatment. Several reports have shown that cells that survive treatment with anti-cancer agents in cancers such as breast, pancreatic, and lung cancers exhibit specific protein expression and molecular mechanisms, correlated with the poor survival rate of patients (58–60). These studies may provide the possibility to maximize the effect of chemotherapy using additional drugs that control key proteins involved in drug resistance.

The characteristics of drug-resistant cancer cells are related to stemness in development, progression, recurrence, and metastasis (61, 62). Cancer stem cells (CSCs) isolated from breast cancer cell lines exhibit drug resistance, and proteomic analyses of these cells suggest new specific markers and therapeutic targets for CSCs (63, 64). Raffel et al. suggested that the importance of targeting leukemic stem cells as the reason for the poor clinical outcomes after treatment for acute myeloid leukemia is due to chemotherapy-resistant cells. Hematopoietic stem/progenitor cells and leukemic stem cell population analysis revealed that IL3RA and CD99 could be markers of leukemic stem cells (65). In addition, pancreatic cancer with a poor prognosis and CSCs are characterized by changes in carbon metabolism and lipid metabolism. Proteomic analysis revealed that the proteins with the highest increase in CSCs were associated with carbon metabolism, and the inhibition of fatty acid synthesis reduced CSC viability, implying a key metabolic pathway regulating CSCs (66). The activity of drug-resistant cancer cells or CSCs interferes with the treatment process, and cancers in which these cells exist are classified as intractable cancers that cannot be treated with conventional anti-cancer drugs. Therefore, for an optimal cancer therapy, it is necessary to identify specific proteins in these cells and identify new diagnostic and therapeutic targets. Table 2 shows a summary of the biomarkers that have been identified in various cancers using proteomics.

**Table 2:** List of representative cancer biomarkers identified using proteomics approaches.

**Type of Cancer**	**Sample Type**	**Method of target discovery**	**MS-based strategy**	**Target**	**Biomarker/ Target Type**	**Features of biomarker**	**References**
Liver (HCC)	Patient's tissue	- Proteomics - Phosphoproteomics	In-solution digestion and LC-MS/MS	PYCR2, ADH1A	- Prognostic	HCC metabolic reprogramming	(48)
Pancreas	Primary Pancreatic Epithelial cells	- Proteomics	In-solution digestion and LC-MS/MS	LKB1	- Prognostic	Regulate pathways associated with glycolysis, serine metabolism, and DNA methylation	(49)
	PDAC cell lines	- Proteomics	In-gel digestion and LC-MS/MS	MAP2	- Prognostic	Proteins involved in microtubule synthesis are upregulated in gemcitabine-resistant cells. Microtubule stabilizing has an effective anti-cancer effect, particularly in MAP2 overexpressed cells.	(58)
Ovary	Patients Tissue	- Proteomics	In-solution digestion and LC-MS/MS	NNMT	- Therapeutic	Central metabolic regulator of CAF differentiation and cancer progression in the stroma	(50)
Breast	Patients Tissue, Breast cancer cell lines	- Proteomics - Metabolomics	in-solution digestion and LC-MS/MS	PYCR1	- Prognostic	The higher the expression of PYCR1, the lower the patient's survival rate. Expression of PYCR1 is involved in acquiring resistance	(59)
	Breast CSCs, Breast cancer cell line	- Proteomics	In-solution digestion and LC-MS/MS	CD66c	- Therapeutic	Proposed as a novel breast CSC marker by modulating the cell viability of CSCs under hypoxic condition.	(64)
	Breast cancer cell lines	- Proteomics	In-solution digestion and LC-MS/MS	NEDD4	- Therapeutic	Presenting as a novel therapeutic target by regulating the expression of ALDH1A1 and CD44, which are characteristic of CSCs	(63)
Lung	EGFR-mutant cell lines	- Proteomics - Phosphoproteomics	In-solution digestion and LC-MS/MS	PI3K/ MTOR	- Therapeutic	In lung cancer resistant to EGFR tyrosine kinase inhibitor, PI3K/MTOR inhibitor was used in combination to overcome resistance	(60)
Myeloid leukemia	Patient-derived AML stem cells	- Proteomics	In-solution digestion and LC-MS/MS	IL3RA, CD99	- Therapeutic	Providing proteomic resources to design leukemic stem cells-targeted therapies by presenting leukemic stem cells-specific markers	(65)

*AML, acute myeloid leukemia; CAF, cancer-associated fibroblast; CSC, cancer stem cell; EGFR, epidermal growth factor receptor; HCC, hepatocellular carcinoma; LC-MS/MS, liquid chromatography-tandem mass spectrometry; PDAC, pancreatic ductal adenocarcinoma; PDX, patient-derived xenografts*.

Therefore, molecular-level studies for cancer treatment applying proteomics are being conducted, and various methods have been proposed. Among them, immunotherapy has become the preferred alternative treatment to a great extent. Unlike chemotherapy and anti-cancer drugs that directly target cancer cells, immunotherapy activates immune cells to induce attacks on cancer cells to eliminate them and controls the tumor microenvironment to enhance the anti-cancer therapeutic effect (67–69). To monitor cancer therapy and prognosis using immunotherapy, it is necessary to utilize appropriate biomarkers. Protein profiling of cancer patients receiving immunotherapy indicates a response to immunotherapy and survival, suggesting the potential of proteomics approaches to discover prognostic biomarkers (70, 71). Moreover, it can be applied to the treatment of cancer patients by providing molecular information on factors causing resistance to immunotherapy (72). These studies suggest that proteomics analysis can be commonly used for cancer patient biomarkers and can help enhance the immunotherapy response. Table 3 organizes the biomarkers and features found using the proteomics approach to immunotherapy.

**Table 3:** List of biomarkers discovered by proteomics approaches to immunotherapy.

**Type of cancer**	**Sample type**	**Biomarker of immunotherapy**	**Description**	**Therapeutic monitoring**	**References**
Liver	Patient's tissue	SLC10A1	Provide predominantly downregulated immune protein cluster between tumor and non-tumor liver	-	(48)
Melanoma	Patient's tissue	MHC	Provide linking melanoma metabolism to immunogenicity and immunotherapy	-	(70)
Lung	Patient's tissue	LAIR1, TIM3	Identify intratumorally collagen that are major source of immune suppression related to murine and human lung cancer	+	(72)
Glioblastoma	Patient's tissue	FAK	Provide glioblastoma factors related to immunotherapy using proteomics/miRNomics	+	(73)
Colon	Patient's tissue	IGF2BP3	Provide a novel information of putative tumor-specific biomarkers that are potentially ideal targets for immunotherapy	-	(26)
Clear cell renal cell carcinoma	Patient's tissue	OXPHOS, PRDX4, BAP1, STAT1	Provide microenvironment cell signatures, four immune-based clear cell renal cell carcinoma	-	(74)
Endometrial carcinoma	Patient's tissue	CDK12	Suggest alternative mechanism for repressing anti-tumor immune response	-	(75)

## Proteomics Approach in Brain Cancer

Glioblastoma multiforme (GBM) is a very aggressive primary brain tumor that presents as heterogeneous malignant types with poor prognosis, high tumor invasiveness, and rapid relapse or progression, resulting in disability during therapy (76–78). Thus, it is urgently needed to identify biomarkers to accurately measure drug response in patients with GBM. In this regard, proteomics analyses have successfully identified alterations in protein expression patterns in GBM.

For example, clinical tissue specimens from patients with high-grade gliomas (glioblastoma) and lower grade gliomas (astrocytoma) have been analyzed with high-resolution iTRAQ labeling quantitative proteomics approach to examine changes in the expression of nuclear proteins. An integrative analysis of both proteomics and transcriptomics data showed that YBX1 is expressed in tumor tissue, and it acts as a regulator of tumor invasion (79). The role of CDH18 in glioma carcinogenesis and its progression examined using proteomic analysis based on their group cohort database. The iTRAQ-based quantitative analysis showed that CDH18 was downregulated in tumor tissue from patients with glioma, and its downstream target, UQCRC2, was down-regulated in tumor tissue from patients with glioma compared to healthy tissue (80). These studies, therefore, defined a target protein for the development of new therapeutic strategies for the treatment of gliomas through proteomic analysis.

Various types of cancers contain a subpopulation known as cancer stem cells that have characteristics similar to normal stem cells, such as self-renewal and the ability to differentiate (81–83). GBM tumors are reportedly composed of heterogeneous types of cells, including a population of stem cell-like cells. The presence of these cells may represent an important therapeutic target because they can cause tumor growth and relapse during therapy (84, 85). To compare the secreted proteins from GBM-derived neural stem (GNS) cells, known cancer stem cells, and normal NSCs, Okawa et al. used a SILAC quantitative proteomics approach that use stable isotope labeling. This study showed that CD9 is enriched in GNS cells (86), indicating that protein and pathways that distinguish GNS cells from NSCs may have value as new biomarkers or candidate therapeutic targets in GBM.

Glioblastoma/astrocytoma can be diagnosed with biomarkers from liquid biopsies, such as plasma, cerebrospinal fluid, and urine, using proteomics. Biomarker candidates for glioblastoma have been found in plasma obtained from glioblastoma patients using SWATH-LC-MS/MS quantitative analysis. Because of this proteomics analysis, eight biomarker candidates for GBM were identified, and among these, LRG1, CRP, and C9 concentrations in plasma were positively correlated with GBM tumor size (87). In addition, Ni et al. have reported the discovery of biomarkers in a model of GBM in which C6 cells are injected into the brain of Wistar rats. They used MRM label-free proteomics to analyze the proteins present in urine over time post-cell injection and successfully identified 109 proteins that changed over time prior to any tumors being visible by MRI (88). Several proteomics studies have discovered biomarkers through the proteomics analysis of cerebrospinal fluid derived from patients with various types of brain tumors such as medulloblastoma (89), pediatric brain tumors (90), CNS lymphoma (91–93), and glioblastoma (94). Therefore, the biomarkers for brain tumors have been identified through proteomics, and the organization of that information led to the recent development of a monitoring system for multiple glioblastomas.

Recently, a glioblastoma phase II clinical trial using personalized immunotherapy failed to prolong patient survival. Despite this, Erhart et al. used a novel approach combining MS-based TMT quantitative proteomics and miRNA sequencing plus RT-qPCR to search for molecules that were related to treatment failure (73). In this way, target proteins and molecules causing therapeutic failure were discovered and analyzed to validate the potential of the novel approach. This strategy could be useful in identifying the factors associated with the failure of other cancer therapies and may pose the basis for the development of new therapies, including cancer immunotherapy.

In a rat xenograft model of human glioblastoma, a survival benefit was observed compared to the untreated control when animals were treated with bacterial carriers that can migrate to the tumor zone where they can induce apoptosis *via* hypoxia-induced expression of the p53 tumor-suppressor in the presence of the pro-apoptotic drug. The proteomics analysis in non-responders using LC-MS/MS revealed the presence of competing mechanisms being pro-apoptotic in the synapse in parallel with drug resistance (95). The proteomics approaches allow predicting a patient's prognosis by observing alterations in proteins that act as survival factors after cancer therapies.

## Extracellular Vesicles as Brain Cancer Biomarkers

Cancer-derived extracellular vesicle (EV) containing DNA, RNA, protein, and lipids expand tumor aggressiveness delivering oncogenes into the circulatory system; thus, affecting the tumor microenvironment and body tissues in patients with cancer. However, cancer-derived EVs have the potential to be used to evaluate glioblastoma tumors as biomarkers (96–98). EVs from glioma expressing constitutively active epidermal EGFRvIII affecting the progression of GBM, including cell infiltration, angiogenesis and regulation of the tumor microenvironment, have been profiled. Using MS, label-free quantitative proteomic analysis in glioma cells, Choi et al. characterized the EVs, including their protein composition, and alterations in regulatory genes. EGFRvIII expressing cells were found to be richer in released extracellular exosomes compared to EGFRvIII-negative cells. The EVs from EGFRvIII expressing glioma cells reportedly contained pro-invasive proteins, such as CD44, CD147, and CD151 (99). This proteomics approach suggests that oncogenic changes in cancer cells can regulate the proteome and provide valuable information for the use of cancer biomarkers based on EVs.

EVs were isolated from the plasma of patients with glioma, and their proteome was analyzed with TMT labeling LC-MS/MS method to show that the EVs from patients with high-grade gliomas contained high levels of SDC1 (100). These data support the notion that high- and low-grade gliomas can be distinguished by examining the proteome of EVs isolated from the plasma of patients and, therefore, represent a useful marker for non-invasive diagnosis of glioma. In another study, a Cavitron ultrasonic surgical aspirator was used to isolate EVs from tumor tissues and fluid in patients with grade IV and grade II-III glioblastomas. Proteomics analysis of the EVs using label-free quantitative LC-MS/MS identified differentially enriched proteins. Among these, CCT2, CCT3, CCT5, CCT6A, CCT7, and TCP1 were increased in the EVs of glioblastoma patients; CCT6A was proposed as a biomarker, as it was associated with decreased survival (101). Therefore, cancer-derived EVs not only play a role in cancer pathology but could also be exploited as biomarker candidates to detect cancer.

Intriguingly, GBM proteomics provided multiple molecular targets. It seems to reflect the difference among sample type, sample state, pretreatment methods, and analytical methods. Table 4 summarizes the molecular targets of brain cancer related to diagnosis, prognosis, and therapy. Although the GBM biomarkers identified are looked diverse, they have been reported to be included in cancer-related pathways such as translation and receptor tyrosine kinase pathways (102, 103). Based on this, it is expected the biomarkers have the potential to function in a common pathway involved in the GBM development. Further Omics research will help reveal the exact protein components included in GBM developmental pathway. To discover reproducible and reliable molecular targets, multi-Omics analysis accompanying data integration must be conducted.

**Table 4:** List of molecular targets in brain cancer identified with proteomics approaches.

**Type of cancer**	**Sample type**	**Method of target discovery**	**MS-based strategy**	**Target**	**Biomarker/ target type**	**Features of biomarker**	**References**
Glioblastoma	Patient's tissue	- Proteomics	In-solution digestion and LC-MS/MS	YBX1	- Prognostic - Therapeutic	Major tumor invasion-regulated proteins	(79)
Glioblastoma	Primary GBM subtypes	- Proteomics	In-solution digestion and LC-MS/MS	CD9	- Therapeutic	Highly expressed in primary GNS cells	(86)
Glioblastoma	Glioma cells	- Proteomics	In-gel digestion and LC-MS/MS	EGFRvIII	- Therapeutic	EGFRvIII expression is associated with pro-invasive proteins through EV profile	(99)
Glioblastoma	Blood	- Proteomics	In-solution digestion and LC-MS/MS	LRG1, CRP, C9	- Prognostic	Concentration in plasma correlated significantly with tumor size	(87)
Glioblastoma	Patient's tissue, Fluid	- Proteomics	In-solution digestion and LC-MS/MS	CCT6A	- Prognostic	CCT6A in EV is associated with induction of expression and amplification and negative survival in glioblastoma	(101)
Glioma	Plasma	- Proteomics	In-solution digestion and LC-MS/MS	SDC1	- Diagnostic	High-grade glioma and low-grade glioma through SDC1 present in EV in the patient's plasma	(100)
Glioma	Patient's tissue	- Proteomics	In-solution digestion and LC-MS/MS	CDH18	- Prognostic	Role of tumor-suppressor	(80)
Astrocytoma	Urine from tumor model	- Proteomics	In-solution digestion and LC-MS/MS	109 proteins	- Prognostic	Protein alteration by date, diagnosis before tumor is seen in MRI	(88)

*EGFR, epidermal growth factor receptor; EV, extracellular vesicle GBM, glioblastoma multiforme; GNS, GBM-derived neural stem; LC-MS/MS, liquid chromatography-tandem mass spectrometry; MS/MS, tandem mass spectrometry*.

## Construction of Cancer Databases

Databases have been created using data obtained from proteomics analyses of various types of cancer, and as a result, novel biomarkers and therapeutic targets have been proposed. The proteomics database is updated by collecting data on the proteome, including protein variations (CanProVar 2.0), extracellular matrix composition (MatrisomeDB), and differentially expressed proteins (dbDEPc 3.0) in cancer based on MS, providing resources for the study of various types of human cancer (104–106). Currently, numerous cancer research-oriented institutions around the world contribute to the integration of Omics data resulting in the construction of databases that can provide information to cancer researchers in a facile manner. The HUPO (Human Proteome Organization) focuses on the analysis of human tumors to identify specific signatures associated with multiple types of cancer and extend the genomic data format in the “Proteomics Standards Initiative” to support proteomics information. Similarly, The CPTAC (Clinical Proteomic Tumor Analysis Consortium) has conducted in-depth proteomics studies to publish key findings in several tumors (107–109), which are available through the CPTAC data portal. In addition, the proteomics data in CPTAC has been integrated with cBioportal, a useful information site for cancer genomics research, to facilitate the easy exploration and integrated analysis of proteomics data sets with clinical and genomic data (110). The LinkedOmics web application has three analytical modules that provide a platform for accessing, analyzing, and comparing cancer multi-omics data within and across cancer types. The database contains multi-omics and clinical data from The Cancer Genome Atlas (TCGA) program, and integrates MS-based global proteomics data generated by the CPTAC on selected TCGA tumor samples (26). CMPD is another database that integrates genomics and proteomics data sets. This database is used to address the complex biological properties of cancer, as it facilitates the identification of cancer-related mutated proteins that are encoded by mutated genes (111). Thus, to obtain more accurate information about cancer, some of the databases incorporate proteomics data into genomic-level studies, providing unified descriptions of cancer mutations at the DNA, RNA, and protein levels through subdivided databases. Various MS-based data sets can be used by researchers worldwide using a storage system as a database (112). In addition, a recently developed tool called ProteomicsDB can analyze various data sets of multiple cells, tissues, and organs by integrating proteomics data with other omics data from programs such as the Human Protein Atlas, which has the aim of mapping all the human proteins in cells (113). The proteomics database information that is available shows that it can be used for basic research, drug discovery, or decision making in the clinic. Studies have suggested that proteomics profiling can be used to investigate the biology of cancer, as well as to screen for and discover molecular biomarkers for the diagnosis, prognosis, and treatment of cancer. Table 5 presents the current proteomics databases.

**Table 5:** Databases containing cancer proteomics data sets.

**Database**	**Database sources**	**Experimental platform**	**Description**	**URL**
CanPro Var 2.0	- 26 cancer types	- Proteomics	Based on functional analysis related to protein interaction, it provides a protein sequence database with efficient interpretation of cancer- and non-cancer-related mutations.	http://canprovar2.zhang-lab.org
cBioportal	- More than 200 cancer genomics data sets from TCGA - Proteomics data from CPTAC (breast, colorectal, and ovarian cancers)	- Genomics - Proteomics	Incorporates genomics and proteomics data from various cancer tissues and links molecular profiles with clinical attributes to support the translation of rich data sets into clinical applications.	https://www.cbioportal.org
CPTAC data portal	- 13 tumor sites - 3,854 samples	- Genomics - Proteomics	A data integration system that systematically identifies cancer-related proteins and provides cancer proteogenomics data and analytical methods.	https://cptac-dataportal.georgetown.edu
CMPD	- 1,008 cancer cell lines - 20 tumor types from TCGA (5,625 cancer samples)	- Genomics - Proteomics	Integrates genomics and proteomics data sets, providing cancer mutation data at the DNA, RNA, and protein levels, and facilitating the identification of cancer-related mutated proteins and resources for translational research.	http://cgbc.cgu.edu.tw/cmpd
dbDEPc 3.0	- 26 cancer types (28 subtypes)	- Proteomics	Database of differentially expressed proteins in cancers with multi-level annotations and drug indications.	https://www.scbit.org/dbdepc3/index.php
HUPO Proteomics Standards Initiative	- Proteomics data including multiple cancer types	- Proteomics	Supports large-scale proteomics projects by comparing multiple tumor types to identify specific signatures and expand genomics data formats.	http://www.psidev.info
jPOSTrepo	- Storage of various proteome experimental data sets (including cancers)	- Proteomics	Public repository for sharing mass spectrometry-based protein data (MS/MS raw and processed data) sets, consisting of a file upload process, a high-quality file management system, and an easy-to-use interface.	https://repository.jpostdb.org
Linked Omics	- 32 cancer types, 11,158 patients from TCGA - Proteomics data from CPTAC	- Genomics - Proteomics - Transcriptomics	A database containing clinical and multi-omics data that integrates global proteomics data for different human cancer types. Three analytical modules that provide a platform for access, analysis, and comparison.	http://www.linkedomics.org
MatrisomeDB	- 15 normal tissues - 6 cancer types	- Proteomics	Proteomics database for the ECM, it enables retrieval of ECM proteomic information from normal and cancer tissues.	http://www.pepchem.org/matrisomedb
ProteomicsDB	- Protein-centric multi-organism data sets (including more than 1,000 cancer cell lines)	- Proteomics - Transcriptomics	Protein database for investigating quantitative mass spectrometry-based proteomics data, including drug-target interaction, RNA sequencing, and cell line survival data, facilitating user data analysis with stored data.	https://www.ProteomicsDB.org

*CPTAC, Clinical Proteomic Tumor Analysis Consortium; ECM, extracellular matrix; HUPO, Human Proteome Organization; MS/MS, tandem mass spectrometry; TCGA, The Cancer Genome Atlas*.

## Recent Trends in Proteomics

The human genome is very complex and is regulated at multiple levels so that genomic information can only be obtained with a variety of Omics methods but not with a single approach. Omics research, including genomics, transcriptomics, proteomics, metabolomics, and epigenomics, has advanced considerably through numerous technological advances and is now capable of providing information at various molecular levels, all of which have contributed significantly to our understanding of biological phenomena (25, 114, 115). In addition, it can provide key information in various diseases, which can then be effectively used in biological, medical, pharmaceutical, and industrial applications. However, since the information provided by each single Omics approach, such as at the gene (DNA or RNA), RNA, protein, and metabolite levels, there are limitations in obtaining comprehensive information on the genome. Each analysis will have variations that depend on the methodology, the equipment, and how the data is integrated, meaning that it is difficult to obtain reproducible, standardized results. The results of independent Omics studies are not sufficient to identify significant correlations in each of the high-level Omics analyses (23, 116–118). Clinically, when using an Omics analysis, there are no standard guidelines for defining a patient's clinical samples due to which they are not well-defined and classified, making it difficult to interpret the analytical results (119, 120). Thus, it has been proposed that a standardized clinical database that integrates the Omics analysis results from each patient needs to be created. To diagnose and treat each disease, researchers have made efforts to obtain new molecular information by reproducing analytical data, integrating databases, and standardizing each analysis step to allow for the production of identical analytical values anywhere in the world. In particular, the cancer research field has actively used Omics technologies, analyzing numerous different types of tumor samples, and using the data to develop new cancer treatments. Accordingly, a lot of information has been released through several consortiums.

The International Cancer Genome Consortium (ICGC) is an organization that provides a forum for the collaboration of cancer genome researchers in the fields of genomics and informatics, and that has systematically analyzed over 25,000 cancer genomes at the multi-omics level for 50 different cancer types (121, 122). These large-scale studies have collected whole genome and exome somatic mutation data from patients with, for example, breast, colorectal, pancreatic cancer and GBM as well as a repertoire of oncogenic mutations that enables the definition of clinically relevant types for prognosis and post-treatment management. They connect current genomic data with newly generated transcriptomic data by linking them with clinical and health information. These big data sets can establish specific criteria and methods to improve patient health, such as cancer prevention strategies, early disease detection, biomarker discovery, diagnosis, and prognosis. In addition, it provides multiple-omics data sources to enable the discovery of novel therapies for cancer patients in clinical trials (121).

The CPTAC aims to understand the molecular mechanisms behind the diversity of cancers using large-scale proteomics and proteogenomics (12). The member institutions have molecularly investigated blood, tumor tissues, and surrounding normal tissues of cancer patients at the gene and protein levels to find proteins that may promote cancer growth and become targets for treatment (123). All clinical information related to cancer patients is provided in the database, and the data are accessible (124). In addition, they have optimized proteomics-based technologies such as sample preparation, peptide extraction, chemical labeling, and MS, and provided experimental guidelines for MS, created new proteomics analyses to identify biomarkers in various cancer types, and provided access to radiology and histopathology data (images, etc.) (110, 125). Recently developed techniques enable the quantification of human proteins from a significantly small amount of sample. For example, Myers et al. developed a highly sensitive proteomics protocol using n-column TMT labeling and multiplexing, providing evidence for the post-transcriptional regulation of gene expression (126). The BASIL, a method for identifying phosphorylation and post-translational modification (PTMs) in relatively few cells lacking sensitivity in phosphoproteomic workflows, has been developed and verified using human pancreatic islet cells (127). These technological developments enable simple and effective quantitative multiplexed proteomics analysis of relatively small amounts of biological or clinical samples. Due to the lack of PTM signature databases, the analysis of signaling pathways generally regulated by post-translational modifications, such as phosphorylation analysis, has been performed using PTM data sets generated by MS at the gene-centric level. Accordingly, a freely available database of PTM signatures has been developed and compared to gene-centric methods in assessing signaling events in cancer cells treated with different agents, targeting signal transduction and cell cycle pathways (128).

The use of artificial intelligence (AI) or machine learning (ML) in combining a large amount of caner proteome and overcome data complexity from different data sources has been highlighted recently. AI can be implemented to create algorithms that increase their performance when certain types of resources or data are provided (129). Several studies have used AI tools to identify novel cancer biomarkers or predict cancer stages (130). Shen et al. used the Boruta algorithm to identify mutant genes involved in vascular invasion from TCGA, National Institute of Health, Medical Research, and AMC databases. A total of 10 genes were identified as vascular invasion-related mutations. Although it is yet to be confirmed whether this mutation can be used for clinical prediction, this study supports that ML can discriminate the gene mutation profile in hepatocellular carcinoma. Another example is connecting microscopy images and proteomics through ML (131). This study adopted a convolutional neural network algorithm to analyze histology from the Cancer Imaging Archive and proteomics datasets from CPTAC. Consequently, the histology-based prediction model accurately distinguishes renal cell carcinomas from normal samples; these predictions are strongly associated with a subset of protein markers. Future studies are required to determine whether this algorithm-based prediction is useful for other types of cancer. In addition, the application of AI to omics can further improve target profiling and integration in cancer and disease diagnosis and biomarker discovery.

To better understand the biomedical characteristics through cell analysis, research is being conducted at the single-cell level. Single-cell analysis has the advantage of identifying the unique characteristics of each cell. Various genome and transcriptome studies have conducted single-cell analysis at the DNA and RNA levels, and the need for proteomic studies at the single-cell level has also been discussed (132, 133). Proteins are fundamental units representing the phenotype and function of cells. Because the protein expression profile of each cell is different, the proteome of a single cell has the potential to not only identify detailed and specific protein expression patterns of a cell, but also understand the biological characteristics of each cell (134). Proteome analysis of bulk samples is used to obtain the proteome information of entire cells, which has limitations in obtaining cell-specific information due to factors such as cell heterogeneity (133, 134). Single-cell proteomics can obtain cell-specific information and identify changes that are specific to a certain cell type. Thus, protein changes can be classified in a more accurate and detailed cell-specific manner without being affected by the cell heterogeneity present in the analysis (135–137). However, for the analysis of single-cell proteomics, a reliable cell separation technique is required, and even when single cell isolation is achieved, the amount of expressed protein is small, hindering the analysis. Various methods have been proposed to overcome these limitations, including methods based on antibodies to quantify proteins at the single-cell level (138, 139). Recently, one of these methods succeeded in identifying more than 1,000 proteins from mouse embryonic stem cells at the single-cell level in an analysis based on mass spectrometry (140). Single-cell proteomics is at its infancy compared to single-cell genomics and transcriptomics, but its importance and necessity are increasing. In the future, with the continuous advancement of methods and technologies, our understanding of the biomedical characteristics of single cells will be extended to the protein level, allowing more direct and accurate information acquisition, including genetic information on prognosis, survival, and diagnosis, and novel biological discoveries in cancer.

## Multi-Omics Approach

Cancer genomics and transcriptomics improve our understanding of cancer development and progression, and may lead to diagnosis, prediction, identification, and verification of cancer biomarkers for treatment (141, 142). Transcriptomics directly analyzes the RNA produced through transcriptional processes from genes contained in biological samples, including protein coding genes, mRNAs, small RNAs, and microRNAs (143). The analysis of the transcriptome in cancer samples is an important means of understanding how the expression of different genetic variants affects cancer. After the onset of cancer, the signaling pathways that play a key role in its biological activity, including progression and metastasis, are generally regulated by PTMs in cancer cells (144), in which components of the proteome and their networks are directly involved, performing key molecular functions in cells or tissues. The type of protein modification determines the function and activity of a protein. Protein modification occurs in various ways depending on the cancer cell cycle, pathological conditions, and microenvironment (145). Transcriptome data do not provide direct information on the proteome activity *in vivo*, thus limiting prognosis prediction and observations of drug responsiveness. On the other hand, proteomics-based protein modification measurements allow the analysis of signaling pathways in cancer cells, prognosis, and drug responsiveness. This means that not only transcriptome markers but also proteome markers are crucial for the establishment and validation of cancer targets and biomarkers. However, the genetic makeup differs between people, which hinders the identification of proteins with individual-specific sequence alterations caused by mutant genes and expressed in cancer. To address this, it would be useful to discover more reliable biomarkers and develop personalized precision medicine for cancer patients by analyzing the transcriptome sequencing data of different patients. Large-scale multi-Omics will allow us to identify the transcripts and proteins that are substantially expressed *in vivo*, explaining different molecular processes in more detail.

Recently, MS-based proteogenomic data obtained from various types of cancer using different methods have been presented, which can be used clinically to reach a deeper understanding of diseases, explain the relationship between a tumor's genome and the proteome, or to resolve tumor heterogeneity associated with clinical outcomes. In addition, several therapeutic alternatives have been proposed through multi-Omics analyses. For example, tumor tissues from diffuse gastric cancer (GC) patients from a young population have been analyzed by both a genomic analysis and a comprehensive proteomics analysis. The results identify the signaling pathways associated with somatic mutations in early-onset GC. This proteogenomic study has improved our understanding of cancer biology and patient stratification in GCs (146). Similarly, in a colon cancer cohort, a proteogenomic analysis has provided new therapeutic opportunities that target signaling proteins, metabolic enzymes, and tumor antigens. Comparisons of tumors and healthy tissues using proteomic and phosphoproteomic analyses have systematically identified colon cancer-associated proteins and phosphosites, suggesting that phosphorylation of retinoblastoma protein, an oncogenic driver, is a new therapeutic target. In addition, the association between reduced levels of CD8 T cells and increased glycolysis in tumors was investigated in this study, indicating that glycosylation is a potential target for overcoming tumor resistance to immune checkpoint blockade (123). Therefore, proteogenomic datasets could be a novel means for the discovery of new biological information and the development of therapeutics. MS-based shotgun proteomics has been used to analyze tumor tissues in patients with cancer and found that the genomic subtype converges with the proteomic subtype in prostate cancer (147). The integration of multi-Omics data such as genomics, epigenomics, or transcriptomics, in combination with proteomics, is more reliable and insightful for the identification of prognostic biomarkers than single Omics data. Table 6 summarizes the cancer biomarkers identified by the multi-Omics approaches and their characteristics.

**Table 6:** List of biomarkers in various cancers identified using multi-omics approaches.

**Type of cancer**	**Sample type**	**Method of target discovery**	**Biomarker / target**	**Type of biomarker**	**Features of biomarker**	**References**
Breast	PDX	- Proteomics - Transcriptomics - Phosphoproteomics	GR	- Prognostic	Glucocorticoid receptor activity is associated with cancer metastasis	(54)
Lung	PDX	- Proteomics - Transcriptomics	Bach1, Ho1	- Therapeutic	Induce lung cancer metastasis	(55)
Colon	Patient's tissue	- Proteomics - Genomics - Phosphoproteomic	Rb phosphorylation	- Therapeutic	Increased proliferation and decreased apoptosis in cancer	(123)
Prostate	Patient's tissue	- Proteomics - Genomics	ACAD8	- Prognostic	Association of low ACAD8 protein abundance with poor outcomes intermediate-risk prostate tumors tissues	(147)
Gastric Cancer (EOGC)	Patient's tissue	- Proteomics - Genomics - Proteogenomics	CTGF, NRP1, RAB23, AXL (Oncogene) SH3GLB2, TNK (tumor suppressor)	- Prognostic	Provides mRNA/protein signatures defining subtypes of gastric cancer	(146)
			ARID1, CDH1, RHOA	- Prognostic	Mutation-phosphorylation association in 80 proteins. Based on this, drug sensitivity can be predicted	
Clear cell renal cell carcinoma (ccRCC)	Patient's tissue	- Proteomics - Genomics - Transcriptomics - Epigenomics	VHL/HIF-1	- Prognostic	Provides evidence for rational treatment selection through large-scale proteogenomic analysis from ccRCC	(74)
Endometrial carcinoma (EC)	Patient's tissue	- Proteomics - Genomics - Transcriptomics	CTNNB1, AURKA, TP53	- Therapeutic	Provides a comprehensive analysis of EC. From this, new therapeutic approaches in EC are suggested	(75)

*EOGC, early-onset gastric cancer; GR, glucocorticoid receptor; Rb, retinoblastoma protein; PDX, patient-derived xenografts*.

In cooperation with the Baylor College of Medicine and the University of Washington Medical School, a proteogenomics database has been developed to explore the potential use of proteogenomics in cancer therapy (148). It has been proposed that these two types of data sets will be used to develop effective therapies and a complete understanding of tumor biology. The analysis of the gene sets obtained from multi-Omics, or other types of integrated studies have become condensed and integrated in order to facilitate the interpretation of multiple enrichment analysis, suggesting a way to obtain data, which is more accurate from cancer proteogenomic data (149). In addition, PepQuery, an integrated proteogenomic method, has been developed that is a quick and easy method for the proteomic validation of new genomic alterations. PepQuery is web-based, allowing for access to MS/MS spectra directly from cancer proteomic studies, and provides standalone program support for MS data (150). In the future, it is expected that, beyond proteomics, the application of proteogenomics in personalized medicine will lead to the development of patient-specific medicine.

The value of multi-Omics technology and datasets lies in the possibility of accurately extracting information to help understand patient-specific molecular complexities. The integration of multi-Omics datasets from cancer enables the large-scale omics analyses of cancer to identify the functional effects of genetic alterations, and to provide evidence for reasonable therapy options derived from tumor pathology. Tumor samples from treatment-naive patients with clear cell renal cell carcinoma were assessed using multi-Omics approaches, including genomic, transcriptomic, proteomic, and phosphoproteomic analyses, thereby confirming each molecular subgroup related to instability. The integration of proteogenomic measurements has been able to uniquely identify dysregulated proteins related to the numerous mechanisms of cells by genetic alterations (74). In addition, multi-Omics analysis was enable to classify protein expression patterns that changed in subtypes of endometrial cancer and suggested a method for maximizing the immunotherapeutic effect by targeting immune cells in these patients (75). Collaboration between PrecisionFDA and NCI-CPTAC identified mislabeled data and applied a process for accurate sample identification to multi-Omics studies so that correct data can be attributed to patients (151).

As such, cancer research using omics is further strengthened, and multi-omics data for translational research is being collected globally. With the discovery of new molecular mechanisms and molecular targets, in-depth multi-Omics analysis of cancer specimens has established networks, capabilities, and expertise at the genome, transcriptome, and proteome levels to improve our understanding of the molecular basis of cancer (152–155). These multi-omics analyses of the disease are taking place in the form of collaborative research worldwide.

## Conclusion and Perspectives

Proteomics provides valuable information in several areas, including protein profiles, protein levels, sites of modification, and protein interactions in pathophysiological conditions. Because of this, cancer proteomics identified clinically applicable, novel biomarkers and therapeutic targets. The proteomics approach in cancer research has investigated molecular mechanisms and provided key information on cancer growth, metastasis, and therapy. Importantly, recent cancer proteome databases are established globally and can be freely accessed and used through the integration with bioinformatics. In this paper, we have reviewed the current state of proteomics in multiple cancers. In the case of cancer, the databases are well-organized; however, various diseases the organization of the database information is suboptimal compared to the cancer research database. To address this shortfall, systematic proteomics approaches should be carried out in a variety of diseases, and appropriate databases should be established to provide disease-related information.

**Figure 2:**
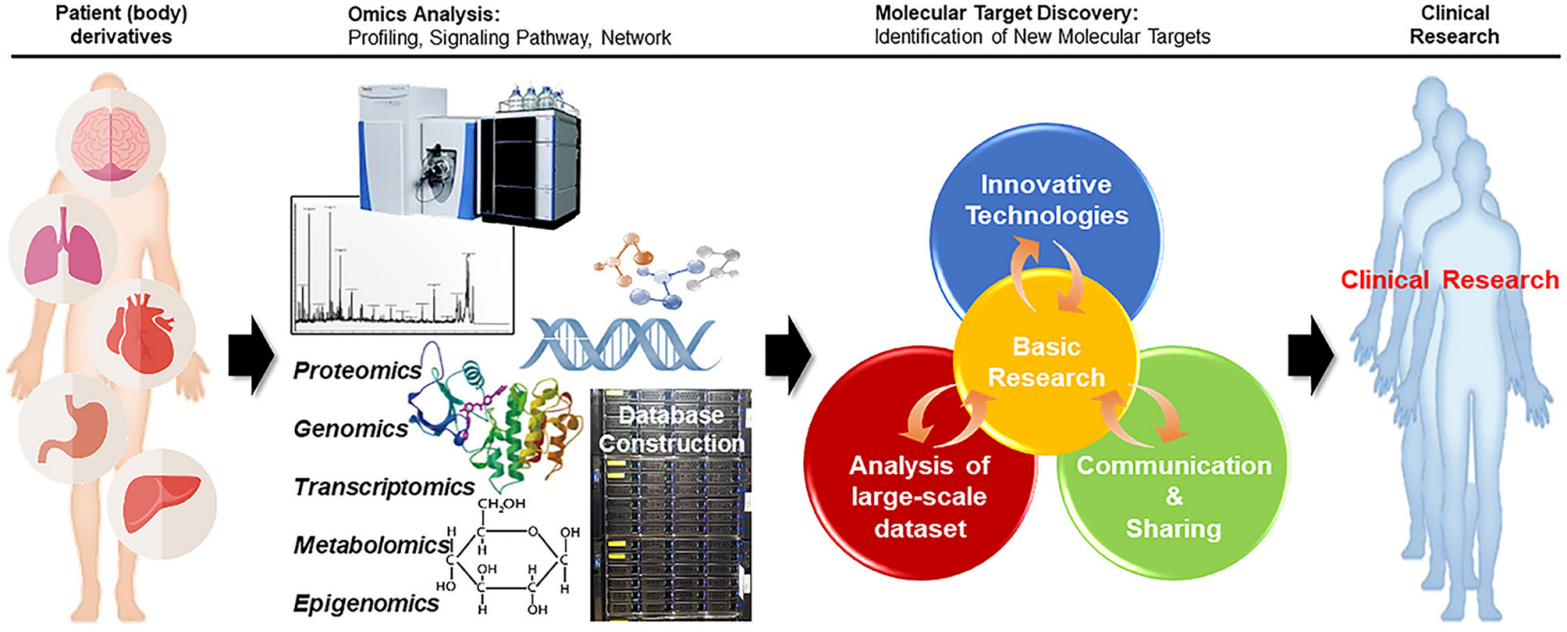
Reverse translational research strategy. In reverse translational research, in-depth multi-omics analysis of cancer specimens from patients can improve our understanding of the molecular basis of cancer, facilitating the discovery of new target molecules. Further clinical research with patients can aid in finding better approaches for the treatment of diseases.

Most Omics technologies aim to enhance cancer therapies, but multi-Omics has opened a new path for cancer-related translational research. As the principal information source for translation research, multi-Omics data are being collected in numerous ways. In the future, it will be possible to reverse the translational research to find molecular targets immediately from patients and to apply them to patients in the clinic using the information collected through the research (Figure 2).

The application of multi-Omics to translation research will take into account not only data collection, integration, and accumulation, but also other aspect including expertise, ethical understanding, communication, administration, and the ability to analyze, interpret, edit and share Omics data across areas. In order to achieve this deeper understanding of the interaction between each Omics data set will be needed.

## Author Contributions

YK and JY conceived the idea and designed the study. H-SJ and SB searched the literature and examined the paper. PS contributed to the idea generation. YK, YS, MS, and JY wrote and revised the manuscript. All authors have read and approved the manuscript.

## Funding

This research was supported by KBRI basic research program through the Korea Brain Research Institute funded by the Ministry of Science and ICT (21-BR-02-08) and by the 2020 Yeungnam University Research Grant (MS). This research was also supported by the National Research Foundation of Korea (NRF) grant funded by the Korea government (2020R1C1C1005500) and by the Dongil Culture and Scholarship Foundation.

## Conflict of Interest

The authors declare that the research was conducted in the absence of any commercial or financial relationships that could be construed as a potential conflict of interest.

## Publisher's Note

All claims expressed in this article are solely those of the authors and do not necessarily represent those of their affiliated organizations, or those of the publisher, the editors and the reviewers. Any product that may be evaluated in this article, or claim that may be made by its manufacturer, is not guaranteed or endorsed by the publisher.

## Glossary

LC-MS/MS, liquid chromatography-tandem mass spectrometry; SILAC, stable isotope labeling by with amino acids in cell culture; ICAT, isotope-coded affinity tag; TMT, tandem mass tag; iTRAQ, isobaric tags for relative and absolute quantitation; MRM, multiple reaction monitoring; SWATH, sequential window acquisition of all theoretical fragment ion spectra; ELISA, enzyme-linked immunosorbent assay; PYCR2, Pyrroline-5-Carboxylate Reductase 2; ADH1A, Alcohol dehydrogenase 1A; CTNNB1, Catenin Beta 1; ALDOA, Aldolase A; LKB1, Liver kinase B1; mTOR. mammalian target of rapamycin; NNMT, N-methyltransferase; ROR1, Receptor-tyrosine-kinase-like orphan receptor 1; Nrf2, nuclear factor erythroid 2–related factor 2; Bach1, BTB domain and CNC homolog 1; Keap1, Kelch-like ECH-associated protein 1; Fbxo22, F-box only protein 22; YBX1, Y-box binding protein 1; CDH18, Cadherin 18; UQCRC2, Ubiquinol-cytochrome c reductase core protein 2; miRNA, MicroRNA; EGFR, Epidermal growth factor receptor; SDC1, Syndecan-1; CCT2, Chaperonin containing TCP1 subunit 2; CCT6A, Chaperonin containing TCP1 subunit 6A; TCP1, T-Complex 1; LRG1, Leucine-Rich Alpha-2-Glycoprotein 1; CRP, C-reactive protein; C9, Complement component C9; CNS, Central nervous system; BASIL, Boosting to amplify signal with isobaric labeling.
